# Potentially toxic metals in irrigation water, soil, and vegetables and their health risks using Monte Carlo models

**DOI:** 10.1038/s41598-023-48489-4

**Published:** 2023-12-01

**Authors:** Muyiwa Michael Orosun, Samuel Nwabachili, Reem F. Alshehri, Maxwell Omeje, Ibtehaj F. Alshdoukhi, Hussein K. Okoro, Clement O. Ogunkunle, Hitler Louis, Fakoya A. Abdulhamid, Stephen Erhonmonsele Osahon, Adamu Usman Mohammed, Emmanuel Olusegun Ehinlafa, Sodiq Omotayo Yunus, Oluwatobi Ife-Adediran

**Affiliations:** 1 Radiation, Health, and Environmental Physics Group, Physics Department, University of Ilorin, Ilorin, Nigeria; 2 Department of Physics, University of Ilorin, Ilorin, Nigeria; 3 Department of Chemistry, College of Science, Taibah University, Medina, Saudi Arabia; 4 Department of Physics, Covenant University, Ota, Ogun State Nigeria; 5 Department of Basic Sciences, College of Science and Health Professions, King Saud Bin Abdulaziz University for Health Science, King Abdullah International Medical Research Center, Riyadh, Saudi Arabia; 6 Department of Industrial Chemistry, University of Ilorin, Ilorin, Nigeria; 7 Department of Plant Biology, University of Ilorin, Ilorin, Nigeria; 8 Department of Chemistry, University of Calabar, Calabar, Nigeria; 9 Department of Applied Geology, Abubakar Tafawa Balewa University, Bauchi, Nigeria; 10 University of California, Berkeley, CA USA; 11 Department of Research Analytics, Saveetha Dental College and Hospitals, Saveetha Institute of Medical and Technical Sciences, Saveetha University, Chennai, India

**Keywords:** Environmental sciences, Environmental chemistry, Environmental impact, Environmental monitoring

## Abstract

Food safety has become a serious global concern because of the accumulation of potentially toxic metals (PTMs) in crops cultivated on contaminated agricultural soils. Amongst these toxic elements, arsenic (As), cadmium (Cd), chromium (Cr), and lead (Pb) receive worldwide attention because of their ability to cause deleterious health effects. Thus, an assessment of these toxic metals in the soils, irrigation waters, and the most widely consumed vegetables in Nigeria; Spinach (*Amaranthushybridus*), and Cabbage (*Brassica oleracea*) was evaluated using inductively coupled plasma-optical emission spectroscopy (ICP-OES). The mean concentration (measured in mg kg^−1^) of the PTMs in the soils was in the sequence Cr (81.77) > Pb(19.91) > As(13.23) > Cd(3.25), exceeding the WHO recommended values in all cases. This contamination was corroborated by the pollution evaluation indices. The concentrations (measured in mg l^−1^) of the PTMs in the irrigation water followed a similar pattern i.e. Cr(1.87) > Pb(1.65) > As(0.85) > Cd(0.20). All the PTMs being studied, were found in the vegetables with Cr (5.37 and 5.88) having the highest concentration, followed by Pb (3.57 and 4.33), and As (1.09 and 1.67), while Cd (0.48 and 1.04) had the lowest concentration (all measured in mg kg^−1^) for cabbage and spinach, respectively. The concentration of the toxic metals was higher in spinach than in cabbage, which may be due to the redistribution of the greater proportion of the metals above the ground tissue, caused by the bioavailability of metals in the aqueous phase. Expectedly, the hazard index (HI),and carcinogenic risk values of spinach were higher than that of cabbage. This implies that spinach poses potentially higher health risks. Similarly, the Monte Carlo simulation results reveal that the 5th percentile, 95th percentile, and 50th percentile of the cumulative probability of cancer risks due to the consumption of these vegetables exceeds the acceptable range of 1.00E−6 and 1.00E−4. Thus, the probable risk of a cancerous effect is high, and necessary remedial actions are recommended.

## Introduction

The soil, though it serves as a membrane for the surface of the earth, also serves as a reservoir for various potentially toxic metals (PTMs). The PTMs are usually transferred from the hydrosphere, biomass, atmosphere, and lithosphere^1–3^. These PTMs are generally generated as a result of both anthropogenic and lithogenic activities. Although PTMs generated as a result of human (anthropogenic) activities are said to be more noteworthy and addressable. These anthropogenic activities include mining, smelting, industrialization, agrochemicals, urbanization, domestic wastes, vehicular activities etc.^4–6^. When the concentration of these PTMs increases above the threshold frequency in the soil, it reduces the quality and productivity of the soil^7^. The anthropogenic activities mentioned do not only affect the soil but also affect water bodies, some of which are used for irrigation. Over the years, an increment in the concentrations of toxic elements and metalloids present in irrigation water has been recorded due to the aforementioned anthropogenic activities. Contaminated irrigation waters when applied to the soil, in turn, cause an increase in the soil concentration which is then passed into the edible parts of a growing plant such as vegetables^8,9^.

Vegetables contain important diet components such as vitamins, protein, minerals, essential trace elements etc. Vegetables also help to buffer both acidic and toxic substances that are usually produced during digestion. The importance of vegetables has made their consumption spike over the years, all around the world. Unfortunately, vegetables grown in contaminated soil potentially store these PTMs in their edible parts, which in turn are passed into the animal bodies and humans alike by ingestion, consequently posing a serious threat to food safety^10^. Food safety has become a serious global concern because of the accumulation of these PTMs in crops cultivated on contaminated agricultural soils^11^. The food web has become a threat to human health, and this is due to the accumulation of these trace elements in the environment. The potential long-term damage caused by the increase of these trace elements present in the vegetables beyond the threshold set by regulations for the entire ecosystem cannot be overemphasized. Regulatory bodies such as the Food and Agriculture Organization (FAO), World Health Organization (WHO), United States Environment Protection Agency (US EPA), and others control the allowable concentrations of the PTMs in food^12,13^. The intake as a food of these defective vegetables can bring about health risks that are classified as carcinogenic and non-carcinogenic. These health risks may include adverse effects on the endocrine, cardiovascular, immune, and urogenital systems, skin lesions and neurological problems^14^. Also, HMs such as Cd, As, Pb, Cr, and Ni have been associated with cancer in various parts of the body which include the heart, kidney, liver, stomach, blood, bone, nervous system, etc.^15^.

In Ilorin, the capital city of Kwara State, Nigeria, every crop is thought to be contaminated with PTMs, thereby making it unsafe for consumption. This is because there has been unprecedented urbanization and industrialization in the State that has led to the introduction of wastewater contaminated with PTMs into most of the irrigation canals. The contaminated water is then used as an irrigation source thereby introducing PTMs into the soil which in turn is passed on to crops that are grown on the soils via soil–plant transfer of PTMs. The food crops consumed in Ilorin are mostly cultivated around the lower Niger River basin where farming activity is prevalent with vegetables being one of the major crops that are grown all-year round. These farming activities are largely supported by the Lower Niger River Basin Development Authority (LNRDA). During the dry season, these vegetable farmers get involved in irrigation as a major source of water.

Several scientists have reported the level of concentration of PTMs in water^16,17^, soils^4,18,19^, and food crops^18,20–22^ from various parts of Nigeria. However, there is a dearth of data on this part of the country (Ilorin) despite its rapid urbanization and industrialization. In fact, besides the works of Ogunkunle et al.^18,17,23^ that explore only the deterministic approach of human health risk assessment models, no other work was found. Unfortunately, the human health risk assessment model is a deterministic analysis model. It contains intrinsic factors that are not certain in the environmental system thereby making it difficult to mirror the unprejudiced situation of pollutants to some extent^4,5,24^. As a result, a stochastic approach using the Monte Carlo simulation (*MCS*) has advertently been utilized appropriately in this study to evaluate more realistic cancer risks attributed to the PTMs. This concept provides researchers with the rare advantage of seeing all the possible outcomes and evaluate the impact of risk. This unquestionably give room for better decision-making in the midst of inherent uncertainties. It accomplishes the risk examination by developing models of probable outcomes by exchanging an array of values (probability distribution) for any factor with uncertainty. In this current work, 10,000 trials were utilized to ascertain the probability (which could be above or below the 95th percentile) of whether a population may be at risk or not.

Hence, this study was carried out to assess the concentrations of arsenic (As), cadmium (Cd), chromium (Cr), and lead (Pb) in the soils, irrigation waters, and the most widely consumed vegetables in Nigeria which are spinach (*Amaranthushybridus*), and cabbage (*Brassica oleracea*), and evaluate the potential dietary health risks to consumers via both stochastic and deterministic human health risk assessment models.

## Materials and methods

### Study area

**Figure 1:**
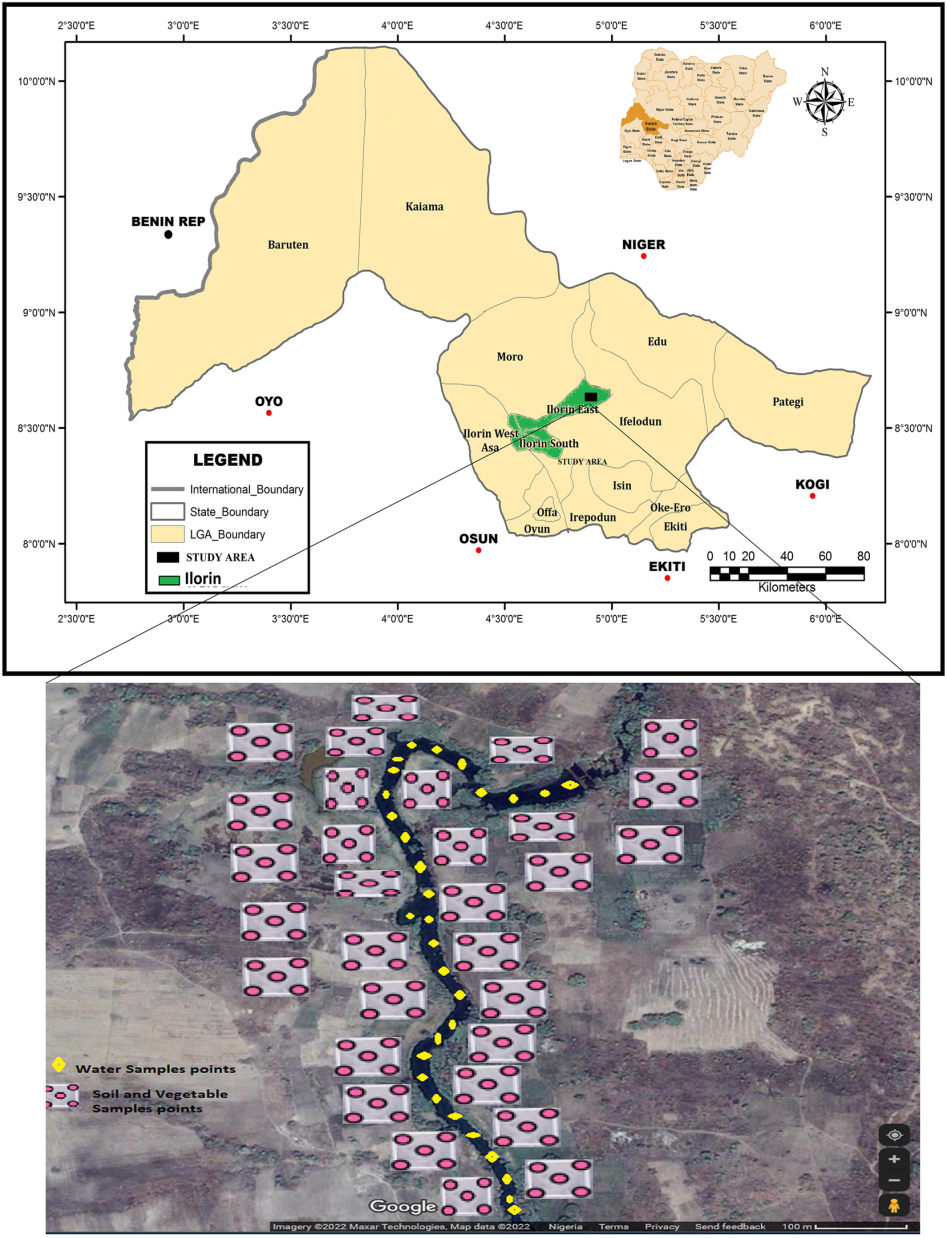
Map and sampling points distribution of the study area.

The study was carried out in Ilorin, the state capital and by far the most populated municipality in Kwara State, Nigeria (Fig. 1). It lies within longitude and latitudes: 8° 20′ N, 4° 25′ E, and 8° 50′ N, 4° 65′ E as shown in Fig. 1. As reported by the National Population Commission of Nigeria (NPC), Ilorin, as of 2011, had a population of 2,748,100 with population density projected to be 74.6 p km^−2^ and a growth rate of + 3.05% per year. Children below 14 years of age make up about 43.5% of the population while 53.3% and 3.2% of the residents are between the ages 15–64 years and over 64 years respectively^4,25^. It was reported by the NPC that the projected population of the study area would be 3,599,800 in 2020. Kwara state, within which the study area is located, has an elevation above sea level of 251 m (equivalent to 823 feet). Its climate is humid wet and dry with an average annual rainfall of 1200 mm^26^. It records a yearly average temperature of 26.2 °C and hottest month temperature of 30 °C in the month of March. Ilorin is located in the south-western region of Nigeria with the rainy and dry months from April to October and November till March, respectively. The dry season follows immediately in November up until March. The study area has crystalline Pre-Cambrian basement complex rocks of the igneous and metamorphic formations. The metamorphic formation is made up of gneiss of the following types: biotite, quartzite augite, banded, and granitic. Pegmatite and vein quartz is comprised of intrusive rocks^27,28^.

### Collection and preparation of samples

#### Soil and vegetables

For archetypal sampling, thirty-one (31) sample sites each covering an area of about 50 × 50 m^2^ as shown in Fig. 1 were mapped out. This covers almost all the farmlands used for the cultivation of vegetables. Surface soil and vegetable samples within the same rooting location were collected from all the sampling points during the harvesting season in March 2021. Five (5) samples of topsoil (0–15 cm) and edible parts (leaves) of the vegetables were collected from each of the mapped thirty-one (31) sample sites giving an X-shape approach, forming a composite sample for the given sample location^29,30^. The composite samples of the soil consist of 155 samples to form 31 composite soil samples, the composite samples of the vegetables comprise 60 cabbages (*Brassica oleracea*) to form 12 composite samples, and 95 spinaches (*Amaranthushybridus*) to form 19 composite samples. The samples from the soil were collected from the rooting region of the vegetable plants, marked for sampling in labeled polyethylene bags weighing about 1 kg using a soil auger. The samples were then taken to the Chemistry Laboratory at the University of Ilorin, to perform the removal of the following: stone fragments, plastic and rubbers, shards, organic matter, and other extraneous particles, in order to make the samples clean^4^. The samples were subjected to air-drying at room temperature for 2 weeks to minimize their moisture content, to get constant dry weight, and finally, prevent any chemical effects from the presence of water in the soil matrices. The samples were grinded to powder using an agate mortar, sieved for homogeneity using a 1 mm mesh size, and then kept in fit-labeled polyethylene vials before digestion. Deionized water was used to wash the vegetable samples in order to rid them of soil particles other contaminants. The edible part of the plant was air-dried under normal room conditions in the Laboratory for 24 h followed by oven drying using an electric oven at 70 °C until a constant dry weight was gotten^4,31^. The marked vegetable plants were collected at each of the points where the previous sampling of soil was done.

Aqua Regia method was adopted for the digestion of the soil and vegetable samples. 1 g of each soil sample was put into to a clean digestion flask followed by the addition of 3 ml and 9 ml of concentrated HNO_3_ and concentrated HCl, respectively^32,33^. The mixture was then subjected to heat until there was no accompanying release of brown fumes, which indicated the liberation of all compounds of nitrogen present in the mixture. This release confirmed that the digestion process was completed. Afterwards, the digests were cooled under normal room conditions and few drops of distilled water were introduced into the mixture and filtered into a standard flask to make a 25 ml volume. This mixture was finally poured out into a polyethylene plastic reagent bottle and the concentrations of Pb, Cr, As and Cd in the digests were analyzed with an Inductively Coupled Plasma Optical Emission Spectroscopy (ICP-OES) following the prEN16174 procedure^34^. Device calibration was ensured using recommended procedures that use standard solutions. Detection limits for Pb, Cr, As and Cd are 0.50, 0.50, 0.05, and 0.07 mg l^−1^, respectively.

In-situ measurements were carried out to determine the pH and electric conductivity (µS cm^−1^) of the soil and water samples.

#### Collection and preparation of water samples

Suitable and clean rubber test bottles were used to collect thirty (30) samples of water from the irrigation canal shown in Fig. 1. Immediately after collection, the water samples were subjected to filtration using 0.45 µm filter membranes. Following the standard method^32^. Aqua Regia was used as the extracting agent for the digestion of the filtrates. Briefly, 100 ml of the collected water samples were measured into a clean dry digestion flask, and 3 ml and 9 ml of HNO_3,_ and HCL, respectively (both concentrated) were added to the samples. Digestion of the samples was then completed after the mixture was heated until every nitrogenous compound is given off as brown fumes. The concentrations of As, Cd, Pb, and Cr in the digests were determined using an Inductively Coupled Plasma Optical Emission Spectroscopy (ICP-OES) instrument following the prEN16174, ECS^34^ procedure as stated earlier.

#### Quality control/quality assurance

The use of analytical grade chemicals as well as the incorporation of sample duplicates and blanks in the analytical procedures of the potentially toxic elements (PTE) were adhered to for accuracy. The equipment were sterilized and prevented from contamination. Pre-cleaning of the glass-wares was properly carried out by soaking all the glass-wares in 6% HCl for 24 h, after which they were thorough rinsed using distilled water and oven-dried at about 105 °C^32,33^. The blanks were analyzed same way as the samples. The percentage recovery greater than 90% was achieved for each of the potentially toxic elements with linear calibration curves (R^2^ of 0.999).

### Bioaccumulation factor (BAF)

The bioaccumulation factor, which is also known as the transfer factor, is defined as the ratio of the concentration of the PTMs found in the vegetables to that which is found in the corresponding soil. BAF values can be calculated using Eq. (1) below

1 $$BAF=\frac{{C}_{vegetable}}{{C}_{soil}}$$

C_vegetable_ denotes the concentration of PTMs (Pb, As, Cr, and Cd), found in the vegetable while C_Soil_ connotes the concentration of PTMs in the soil. In the food chain, one of the main routes that lead to the exposure of humans to these PTMs is the soil-to-plant transfer. A BAF value of less than 1 means that the vegetables absorbed (but did not accumulate) the toxic metal from the soil. BAF values that are greater than one, indicate that the plant absorbed and accumulated the that the toxic element from the soil^4,35^.

### Exposure evaluation

Evaluation of some exposure indices such as the enrichment factor (EF) and Modified Pollution Index (MPI) was used in the assessment of the degree of contamination of the soil, and the irrigation water.

#### Enrichment factor (EF)

To evaluate the enrichment factor (EF), we use the formula given below;

2 $$EF=\frac{{C}_{i}}{{C}_{ref}}$$

where C_i_ = concentration of PTMs in target (soil) while C_ref_ = concentration of HMs given by regulatory bodies such as WHO, FAO, and USEPA. The classification of the results, according to Mokhtarzadeh et al*.*^36^, is as follows: EF < 2 implies that the enrichment is minimal; EF (2–5) implies that the enrichment is moderate; EF (5–20) means that the enrichment is significant; EF (20–40) implies that the enrichment is very high, and EF > 40 implies extremely high enrichment.

#### Modified Pollution Index (MPI)

Equation (3) below gives the formula that is used to evaluate the MPI where EF_mean_ represents the mean of the EF for the PTMs being studied while EF_max_ represents the maximum value of the EF.

3 $$MPI= \sqrt{\frac{{({EF}_{mean})}^{2}+{({EF}_{max})}^{2}}{2}}$$

MPI is a suitable and valid approach to characterize the extent or magnitude of PTM contamination for a given sample of vegetables. The following are the terms used for MPIs based on values:

An MPI that is less than 1 implies, not contaminated; MPI between 1 and 2 suggests slight contamination; MPI between 2 and 3 reveals moderate contamination; MPI between 3 and 5 means significant contamination; MPI greater than 5 and less than 10 implies severe contamination. Finally, MPI greater than 10 implies extreme contamination^36^.

### Health risk assessment

The following steps are important in the evaluation of health risk: assessment of toxicity/dose–response, exposure evaluation, hazard identification, and risk characterization. The risk assessment is a technique which is regularly employed in the assessment of the health effect of due to the exposure of humans to various trace elements^13,24^. Hazard Identification is carried out in order to study the concentration level, and spatial distribution of pollutants present in a particular site. In order to carry out exposure assessment, the population’s Average Daily Intake (ADI) through the ingestion of the trace elements was calculated exposure assessment was carried out. Toxicity resulting from the exposure intensity of the HMs was evaluated by Dose–Response (the response of the body system to a particular amount of trace element) assessment. The slope factor (SF) and reference dose (RfD) which represent a carcinogenic potency factor and a non-carcinogenic threshold respectively, were used as toxicity indices as shown in Table 1. To predict the non-cancerous health risks as well as the probability of cancer development for the populace, the following equation was used.

**Table 1:** Exposure parameters for calculating cancer and non-cancer health risks to humans^5,24,31,37^.

Risk exposure factors	Values	Symbols	Units
Exposure duration	55	ED	Years
Daily vegetable intake	65	DVI	g person^−1^ day^−1^
Body mass	70	BM	kg
Conversion factor (fresh to dry weight)	0.085	Cf	
Exposure frequency	365	EF	Day year^−1^
Time of exposure	ED X 365	TA	Days
Ingestion reference dose	Pb (0.0035), As (0.0003), Cd (0.001), Cr (0.003)	RfD	mg kg^−1^ day^−1^
Ingestion carcinogenic slope factor	As (1.5), Pb (0.0085), Cr (5 × 10^–1^), Cd (3.8 × 10^–1^)	SF	(mg/kg/day)^−1^

The ADI by ingestion of vegetables was calculated using Eq. (4) below;

4 $${ADI}_{inj-veg}=\frac{{C}_{v}\times IngRv \times EF\times ED}{BW\times AT}$$

where ${C}_{v}$ is the concentration of PTMs in the vegetable, BW is the exposed person’s body weight, ED is taken as the duration of exposure in years, AT is expressed in days as the period over which the dose is averaged, EF represents the frequency of exposure in day/year, and IngRv is the ingestion rate of the vegetable. The parameter used is given in Table 1 below.

#### Assessment of non-cancer health risks

The ratio of the estimated ADI to the RfD of a particular PTM results in the target hazard quotient (THQ) which is used for non-carcinogenic risk assessment^24^. To calculate the THQ, the formula given in Eq. (5) is used. The RfD is the maximum oral dose that is allowable for a toxic substance RfD is also said to be the chronic reference dose for a single PTM (mg kg^−1^-day^−1^), while ADI is the average daily intake of a single toxic element.

5 $$THQ=\frac{ADI}{RfD}$$

where ADI is the average daily intake of a single chemical and RfD is the chronic reference dose for the element (mg kg^−1^-day^−1^)^24^. A HQ is greater than 1 indicates a high probability of adverse health effects on the exposed persons. For HQs less than 1, there is no possibility of adverse health effects^24^. The Hazard Index (HI), which is expressed as the total sum of HQ, is calculated for the different exposure pathways using Eq. (6)^15,24,38^. HI is used to aggregate the human health risks as a result of exposure to more than a single PTM, the hazard index (HI) was established^24^.

6 $$HI=\sum HQ$$

#### Assessment of cancer risk

To provide the risk index or calculate the probability of a certain populace developing cancer of any kind after being exposed to a carcinogen over a projected lifetime, the carcinogenic risk assessment is carried out^24,37^. The incremental lifetime cancer risk (ILCR) that projects the likelihood of an exposed individual person developing malignant cells over some time is evaluated using Eq. (7)^24^.

7 $$\mathrm{ILCR}=\mathrm{ADI }\times \mathrm{ SF}$$

where SF (mg kg^−1^ day^−1^) represents the cancer slope factor and ADI is as defined earlier. ILCR values below 1 × 10^–6^ are generally regarded as safe and thus, posing no cancer risk to humans, while greater than 1 × 10^–4^ is largely assumed to be high and thereby posing a higher cancer risk. Hence, the tolerable range for the ILCR is between 1 × 10^−4^ and 1 × 10^–6^.

### Monte Carlo Simulation (MCS) using ORACLE crystal ball software

Monte Carlo simulation is a mathematical technique that is used to evaluate the probable outcomes of any event with uncertainties. This technique is the most widely used approach that accommodates the uncertainties, complexity, and variability linked with many risk-related problems particularly the ones that affect human safety and the ecosystem^4,39–41^. The National Academy of Sciences and USEPA^42,43^ have acknowledged Monte Carlo simulation as a means of appraising variability and uncertainty in health risk evaluations. This is because it presents a quantitative approach to evaluate the probability distributions for health risks within the legitimacy of the risk assessment model^40,41,44^ and offers additional information for decision-making related to human health and environmental protection^45^. Decisions regarding the needs for mitigation or remediation is a very critical task involving the analysis of risks. Decision-makers frequently come across variability, obscurities, and uncertainties in risk analysis. The daily rate of ingestion of the toxic substance (i.e. Cd, Cr, As, and Pb) by individuals that are exposed, their estimated body weight (70 kg was adopted for this study), the quantity of PTMs in the collected samples and their corresponding cancer slope factors are all causes of uncertainty that complicates the appraisal of risk assessment. While underestimation of the cancer risks can lead to avertible radiological health effects on the inhabitants, its overestimation can lead to the waste of scarce resources on needless remediation. Health risk evaluation using the assessment models outlined earlier in "Health risk assessment", is a deterministic approach that underestimates or overestimates the actual risk^5,46^. Consequently, a stochastic method using Monte Carlo simulation (*MCS*) has been used properly in this current work to assess more realistic risks related to the PTMs (Cd, Cr, As, and Pb) present in the vegetable samples. This concept provides researchers with the rare advantage of seeing all the possible outcomes and evaluate the impact of risk. This unquestionably give room for better decision-making in the midst of inherent uncertainties. It accomplishes the risk examination by developing models of probable outcomes by exchanging an array of values (probability distribution) for any factor with uncertainty. The Monte Carlo simulation then calculates the outcomes numerous times (10,000 trials were used), expending several arbitrary values from the probability functions on each occasion. The Oracle Crystal Ball software version 11.1.2.4.850 was used to perform the Monte Carlo simulation.

### Ethical approval

All authors have read, understood, and have complied as applicable with the statement on "Ethical responsibilities of Authors" as found in the Instructions for Authors. Experimental research and field studies on plants, including the collection of plant material, complied with relevant institutional, national, and international guidelines and legislation. Permissions were also obtained from the University of Ilorin Ethical Review Committee that permits the collection of the plant samples.

## Results and discussion

### Levels of PTMs and physiochemical parameters of the soil samples

Table 2 below presents the descriptive statistics of the concentration of the PTMs (As, Pb, Cr, Cd) in the topsoil samples. The pH of the soil ranged between 7.00 and 8.60 with a mean value of 7.64. The mean soil pH value is a little greater than 7.00 implying that this soil is a little basic. The electric conductivity (EC) had a minimum value of 46.8 µS cm^−1^ and a maximum value of 64.3 µS cm^−1^ with a mean value of 58.35 µS cm^−1^.These values fall below the W.H.O. and F.A.O. (Food and Agricultural Organization) recommendations. EC range of between 400 and 600 µS cm^−1^ which implies that the dissolved salt in this soil was not excessive. The concentration for As ranged between 8.97 and 18.23 mg kg^−1^ with a mean concentration of 13.23 mg kg^−1^. This is greater than the world average of 6.83 mg kg^−1^ by 1.94 times (almost twice). The max value of As (18.23 mg kg^−1^) was still in the range of the Maximum Allowable Concentration (MAC) which has a value range of 15–20 mg kg^−1^. The mean value is still in the range of the TAV (10–65 mg kg^−1^). Similarly, Pb recorded a maximum concentration of 23.63 mg kg^−1^, a minimum concentration of 13.31 mg kg^−1^ and a mean concentration of 19.91 mg kg^−1^. According to Table 3, the mean concentration for Pb is greater than the concentration limit for soil, which is 2.0 mg kg^−1^ according to WHO^47^. Cr had a maximum concentration of 91.73 mg kg^−1^, a minimum concentration of 71.32 mg kg^−1^ and a mean concentration of 81.77 mg kg^−1^. Cd had a maximum concentration of 4.72 mg kg^−1^ and a minimum value of 2.05 mg kg^−1^ with an average concentration of 3.25 mg kg^−1^ which is greater than the WHO (i.e. 0.48 mg kg^−1^) for Cd^47^.

**Table 2:** Statistical distribution of concentration of PTMs in soil samples.

Statistics	pH	EC (µS cm^−1^)	As (mg kg^−1^)	Pb (mg kg^−1^)	Cr (mg kg^−1^)	Cd (mg kg^−1^)
Min	7	46.8	8.97	13.31	71.32	2.05
Max	8.6	64.3	18.23	23.63	91.73	4.72
Mean	7.67	58.35	13.23	19.91	81.77	3.25
Median	7.55	58.45	14.21	20.4	83.52	3.12
SD	0.53	5.16	2.82	3.21	6.74	0.78
CV	0.069	0.088	4.69	6.2	12.13	4.17
WA	–	–	6.83	27.00	59.50	0.41
MAC	–	–	15–20	20–300	50–200	1–5
TAV	–	–	10–65	50–300	50–450	2–20

*WA world average, *TAV trigger action value, *MAC maximum allowable concentration in soil^48–50^.

**Table 3:** Recommended limit for selected PTMs in irrigation water, and soil for agriculture.

	Matrix	Recommended limit for the selected PTMs					
		As	Cd	Cr	Pb	EC (µS cm^−1^)	pH
WHO	Irrigation water	0.1	0.01	0.1	5.0	400–600	6.5–8.5
WHO	Soil	**–**	0.48	11.0	2.0	**–**	**–**
WHO	Vegetables	**–**	0.2	2.3	0.3	**–**	**–**
FAO	Irrigation water	**–**	**–**	**–**	**–**	400–600	6.5–8.4

^51–53^.

### Levels of PTMs in the irrigation water sample

Table 4 shows the summary of the concentration of PTMs (As, Pb, Cr, and Cd) in the irrigation water sample. It can be seen that the water sample had a pH level of between 6.5 and 7.6with an average of 7.18. This, is in the WHO and FAO pH range of 6.5–8.5 and 6.4–8.4, respectively. This goes to show that the irrigation water is neutral i.e. not acidic, nor basic. The electrical conductivity (EC) had a value between 365 and 385 µS cm^−1^. This is in the WHO and FAO accepted level of 400–600 µS cm^−1^ as shown in the Table 3. This means that the level of dissolved salt in the water is below the normal standard thereby making the pH to be in the level it is. Electrical conductivity is used to assess the level of salinity. If the level of the EC is beyond the accepted level, this means that the salinity is beyond acceptable as this will increase the EC of the soil, thereby causing it to have an increase in the amount of dissolved salt which leads to over-flocculation of the soil. As, Pb, Cr, and Cd recorded minimum concentrations of 0.11, 1.11, 1.02, and 0.1 mg kg^−1^, maximum concentrations of 1.31, 2.51, 1.02, 2.51 mg kg^−1^ and mean concentrations of 0.85, 1.65, 1.87, 0.2 mg kg^−1^, respectively. The mean concentration of As (0.85 mg kg^−1^) was found to be 8.5 times greater than the WHO acceptable limit of 0.1 mg kg^−1^. Pb had a mean concentration of 1.65 mg kg^−1^ i.e. 3.03 times less than the accepted WHO threshold value of 5.0 mg kg^−1^. On the other hand, Cr had a mean concentration that is 18.7 times greater than the WHO accepted threshold of 0.1 mg kg^−1^. Finally, Cd had a mean concentration that is 20 greater than the WHO accepted value (0.01 mg kg^−1^) (see Table 3). According to Maleki et al*.*^54^, the concentration of these metals in the irrigation water may increase their level in the soil which in turn may cause the metal uptake by the vegetables to upsurge thereby leading to an increment in their concentration in the vegetable and vice versa. The osmotic activity carried out by the vegetable in obtaining water that is already contaminated with these PTMs and from the soil is believed to lead to the bioaccumulation of PTMs in the vegetables.

**Table 4:** Descriptive statistics of the PTM concentration in irrigation water samples.

Statistics	pH	EC (µS cm^−1^)	As (mg kg^−1^)	Pb (mg kg^−1^)	Cr (mg kg^−1^)	Cd (mg kg^−1^)
Min	6.5	365	0.11	1.11	1.02	0.1
Max	7.6	385	1.31	2.51	2.51	0.31
Mean	7.18	377.67	0.85	1.65	1.87	0.2
Median	7.3	380	1.03	1.51	1.92	0.2
SD	0.32	6.71	0.42	0.45	0.51	0.07
CV	22.44	56.28	2.02	3.67	3.67	2.86

### Levels of PTMs in vegetable samples

**Figure 2:**
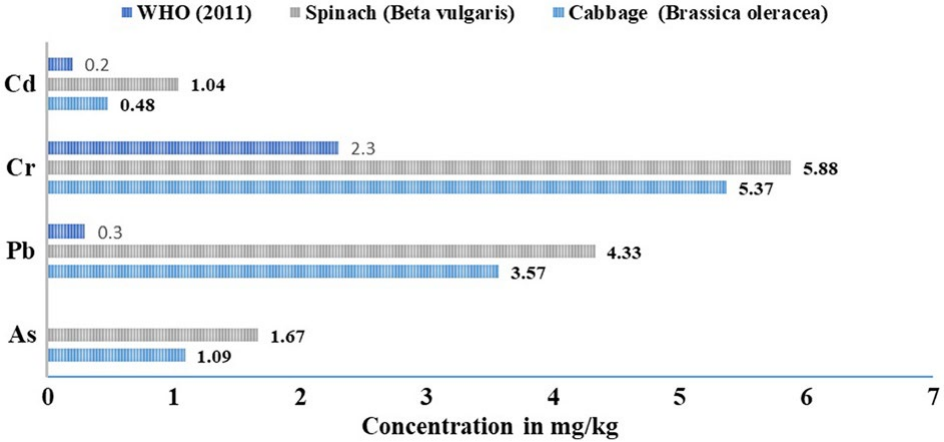
Mean concentrations of PTMs in the vegetable samples.

Table 5 below shows the concentration of PTMs found in the vegetable samples collected from the farms. For cabbage, the concentrations of As, Pb, Cr and Cd range from 0.57 to 1.5, 2.97 to 4.21, 3.64 to 6.56, and 0.24 to 0.63 mg kg^−1^ respectively, with an estimated average value of 1.09, 3.57, 5.37, 0.48 mg kg^−1^. Similarly, for spinach, the concentrations of As, Pb, Cr, and Cd range from 1.02 to 2.11, 1.24 to 4.89, 1.83 to 7.21 and 0.41 to 1.69 mg kg^−1^ with corresponding means of 1.67, 4.33, 5.88 and 1.04, respectively. It was observed that the estimated average value of Cd found in the spinach and cabbage was respectively 5.2 and 2.4 times beyond the WHO^47^ threshold value (0.2 mg kg^−1^) of Cd that is recommended to be present in the vegetable sample. Similarly, the mean concentration of Cr in the spinach and cabbage was 2.5 and 2.3 fold greater than the concentration threshold placed by WHO^47^. The mean concentration of Pb found in the spinach and cabbage was very much higher (i.e. 14.4 and 11.3 times respectively) than the provided concentration threshold of Pb recommended by WHO (0.3 mg kg^−1^) (see Table 3). Recall that Cd at a high level of concentration causes cancer, hypertension and Anaemia, and prolonged exposure to enhanced doses of Cr may lead to deleterious effect in sensitive organs such as kidney and liver. Low exposure to Pb can harm the central nervous system particularly those of infants and children, protracted kidney disease, blood pressure, and cancer^5^. It can be observed from Fig. 2, that the toxic element’s concentrations were higher in spinach than in cabbage. This revelation is in agreement with the findings of Ahmed et al.^10^, Maleki et al.^54^, and others where the higher concentrations were attributed to deposits on the leaves of spinach during irrigation as well as root uptake from soil (absorbed by root hairs and translocated to leaves). Additionally, the variation of the toxic elements in the spinach and cabbage, is dependent on the physiochemical nature of the soil and the vegetable's capacity to absorb each toxic chemical which is greatly affected by environmental factors, planting and farming methods, specie of plant, climatic conditions, etc. The bioaccumulation factor (BAF) also referred to as the transfer factor, was evaluated to assess the mobility of the potentially toxic metals in the soil–plant system. The BAF values for cabbage range between 0.18 (Pb) and 0.07 (Cr), and ranged between 0.23 (Cd) and 0.07 (Cr) for spinach. While these values are all less than 1, implying that the toxic metal was only absorbed by the vegetables but not accumulated, it was observed that the BAF values for spinach were higher than the values for cabbage for all the metals. This corroborates the fact that spinach poses a higher risk than cabbage.

**Table 5:** Statistical distribution of PTMs concentration in the vegetable samples.

Vegetable	Statistics	As	Pb	Cr	Cd
Cabbage (*Brassica oleracea*) (n = 60)	MIN	0.57	2.97	3.64	0.24
	MAX	1.50	4.21	6.56	0.63
	MEAN ± STDV	1.09 ± 0.27	3.57 ± 0.49	5.37 ± 0.97	0.48 ± 0.14
	BAF	0.08	0.18	0.07	0.15
Spinach (*Amaranthushybridus*) (n = 95)	MIN	1.02	1.24	1.83	0.41
	MAX	2.11	4.89	7.21	1.69
	MEAN ± STDV	1.67 ± 0.43	4.33 ± 0.72	5.88 ± 1.53	1.04 ± 0.62
	BAF	0.13	0.22	0.07	0.32

### Pollution evaluation

The enrichment factor (EF) of the understudied PTMs found was analyzed and presented in Table 6. As had a least value of 1.31, highest value of 2.67, and a mean value of 1.94. Pb had a minimum value of 0.49, a maximum value of 0.88, and a mean value of 0.74. Cr had a least value of 1.198, highest value of 1.542, and a mean value of 1.37. Cd had a minimum value of 5, a maximum value of 11.51, and an average value of 7.93. The enrichment factor is very important in determining the level of contamination of the soil. Recall that, EF < 2 implies that the enrichment is minimal; EF (2–5) implies that the enrichment is moderate; EF (5–20) means that the enrichment is significant; EF (20–40) implies that the enrichment is very high, and EF > 40 implies extremely high enrichment. From Table 6, it can be said that the soil was more contaminated by Cd. The levels of EF for As, Pb, and Cr are less than 2 implying minimal or insignificant enrichment. While the EF values of Cd fall between 5 and 20 signifying significant enrichment.

**Table 6:** Pollution evaluation indices.

PTMs	As	Pb	Cr	Cd
EF	1.94	0.74	1.37	7.93
MPI	2.33	0.81	1.46	9.88

The result of the modified pollution index (MPI) is presented in Table 7. As had a pollution index of 2.33, Pb had 0.81, Cr had 1.46, and Cd had 9.88. This implies that the soils were moderately polluted by As and Cr since their MPI values fall between 1 and 3. Pb whose value is 0.81 implies minimal or insignificant pollution by lead. Finally, Cd had a value of 9.88, a considerably high value, a value that is so close to 10. This also reveals that the vegetables would be severely contaminated with Cd. This implies that the populace is at risk of Cd-related diseases. Note that when cadmium is introduced into the body, it is transferred into the bloodstream by erythrocytes. Cd ends up accumulating in the kidneys, livers, and gut. This is because of its slow excretion. Exposure to Cd can lead to myriad deleterious health effects including pulmonary edema, osteomalacia, testicular damage, damage to the hemopoietic and adrenals system, and hepatic/renal dysfunction. Occupational Cd is linked with lung, breast, prostate, pancreas, urinary bladder, and nasopharynx cancers^55–57^. This means that a lot of attention needs to be paid to by the authorities to the introduction of cadmium into the irrigation water.

**Table 7:** Average daily intake (ADI) of PTMs from the vegetables (× 10^–3^).

Vegetable	Stat	As	Pb	Cr	Cd
Cabbage (*Brassica oleracea*)	MIN	0.53	2.76	3.38	0.22
	MAX	1.39	3.91	6.09	0.59
	MEAN	1.01	3.32	4.99	0.45
	STDEV	0.23	0.44	0.86	0.12
Spinach (*Beta vulgaris*)	MIN	0.95	1.15	1.70	0.38
	MAX	1.96	4.54	6.70	1.57
	MEAN	1.55	4.02	5.46	0.97
	STDEV	0.40	0.67	1.42	0.58

### Human health risk assessment

Table 7 shows the average daily intake (ADI) of PTMs from vegetables analyzed. For cabbage, As recorded a maximum value of 1.39 E−3, a minimum value of 0.53 E−3, and a mean value of 1.01 E−3. Pb recorded a maximum value of 3.91 E−3, a minimum value of 2.76 E−3, and a mean value of 3.32 E−3. Cr had a maximum value of 6.09 E−3, a minimum value of 3.38 E−3, and a mean value of 4.99 E−3. Cd had a maximum value of 0.59 E−3, a minimum value of 0.22 E−3, and a mean value of 0.45 E−3. Spinach (*Amaranthushybridus*) As recorded a minimum value of 0.95 E−3, a maximum value of 1.96 E−3, and a mean value of 1.55 E−3. Pb recorded a minimum ADI of value 1.15 E−3, a maximum value of 4.54 E−3, a mean value of 4.02 E−3 Cr had a minimum value of 1.70 E−3, a maximum value of 6.70 E−3, and a mean value of 5.46 E−3. Cd had a maximum value of 1.57 E−3, a minimum value of 0.38 E−3, and a mean value of 0.97 E−3. From the recorded mean values for cabbage (*Brassica oleracea*), it was found that Cr was taken in more, while Cd was least taken in by the populace.

For spinach (*Amaranthushybridus*) the mean values of the ADI also confirm that the dietary intake of Cr was highest, while Cd was the least.

The hazard quotient (HQ) of the PTMs understudy in the vegetables is shown in Table 8. For cabbage, As recorded a maximum HQ of 4.64, a minimum Hazard quotient of 1.76, and a mean value of 3.37. Pb had a maximum HQ of 1.12, a minimum of 0.79, and a mean of 0.95. Cr had a maximum value of 2.03, a minimum value of 1.13, and a mean value of 1.66. Cd had a maximum value of 0.59, a minimum value of 0.22, and a mean value of 0.45. For spinach (*Amaranthushybridus*) Ashad a maximum value of 6.53, a minimum value of 3.16, and a mean value of 5.17. Pb had a maximum of 1.29, a minimum value of 0.33, and a mean value of 1.14. Cr had a maximum value of 2.23, a minimum value of 0.57, and a mean value of 1.82. Cd had a maximum value of 1.57, a minimum value of 0.38, and a mean value of 0.97. The Hazard Index (HI) for cabbage ranges between 8.38 and 3.90, with an average value of 6.43, while HI for spinach ranges between 11.62 and 4.44, with an average value of 9.10. Expectedly, the HI value of spinach is higher than that of cabbage. According to USEPA^24^, HI values, less than 1 (< 1) do not have a probable non-cancerous effect, while values greater than 1 (> 1), have a probable non-cancerous effect. This implies that spinach posesa higher non-carcinogenic risk. Most important is the fact that both the spinach and cabbage have HI values high than 1 which is the standard set by USEPA^24^. This follows that the general populace is at greater risk of non-carcinogenic risks from consuming these vegetables. From the mean values of the PTMs found in all the vegetable samples collected, it is observed that As is the chief contributor to non-cancer toxic risk followed by Cr, Pb, and then Cd.

**Table 8:** Hazard quotient (HQ) and Hazard Index (HI) of PTMs in the vegetables.

Vegetable	Stat	As	Pb	Cr	Cd	HI
Cabbage (*Brassica oleracea*)	MIN	1.76	0.79	1.13	0.22	3.90
	MAX	4.64	1.12	2.03	0.59	8.38
	MEAN	3.37	0.95	1.66	0.45	6.43
	STDEV	0.77	0.12	0.29	0.12	1.30
Spinach (*Beta vulgaris*)	MIN	3.16	0.33	0.57	0.38	4.44
	MAX	6.53	1.29	2.23	1.57	11.62
	MEAN	5.17	1.14	1.82	0.97	9.10
	STDEV	1.33	0.19	0.47	0.58	2.57

Similarly, from Table 9, the estimated incremental lifetime cancer risk (ILCR) was observed to have the highest value of 5.39E−3 and the lowest value of 2.58E-3, and an average value of 4.21E−3, for the Cabbage while the minimum, maximum and mean values of the ILCR for the spinach are 2.42E−3, 6.93E−3, and 5.46E−3 respectively. This also reveals that spinach poses a higher carcinogenic risk than cabbage. As previously mentioned, ILCR values that exceeds 1.00E−4 are regarded high and deplorable as they are adjudged to pose greater cancer risks, while the ILCR values within the lower boundary of 1.00E−6 are considered not to pose any cancer risk to humans. Consequently, the established acceptable cancer risks range is between 1.00E−4 and 1.00E−6. This follows that the estimated ILCR for both spinach and cabbage exceeds the threshold values provided by USEPA^24^. Hence, the probable risk of a cancerous effect is high and the public should be cautious as the risk level for both vegetables falls in Level VII (High Risk) (see Table 10). Thus, the attention of the appropriate authorities is needed and necessary remedial action is recommended.

**Table 9:** Incremental lifetime cancer risk (ILCR) of PTMs from the vegetables (× 10^–3^).

Vegetable	Stat	As	Pb	Cr	Cd	ILCR
Cabbage (*Brassica oleracea*)	MIN	0.79	0.023	1.69	0.08	2.58
	MAX	2.09	0.033	3.045	0.22	5.39
	MEAN	1.52	0.028	2.49	0.17	4.21
	STDEV	0.35	0.004	0.43	0.045	0.83
Spinach (*Beta vulgaris*)	MIN	1.42	0.0097	0.85	0.14	2.42
	MAX	2.94	0.039	3.35	0.596	6.93
	MEAN	2.33	0.03	2.73	0.37	5.46
	STDEV	0.60	0.006	0.71	0.22	1.54

**Table 10:** Levels and values of assessment standards according to Haque et al.^57^ and Li et al.^58^.

Risk levels	Range of risk	Acceptability
Level I	< 10^–6^	Extremely low risk: accept the risk level
Level II	10^–6^, 10^–5^	Low risk: not worry about the risk
Level III	10^–5^, − 5 × 10^–5^	Low-medium risk: not wary of the risk
Level IV	5 × 10^–5^, − 10^–4^	Medium risk: care about the risk
Level V	10^–4^, − 5 × 10^–4^	Medium–high risk: worry about the risk and be eager to participate in it
Level VI	5 × 10^–4^, − 10^–3^	High risk: show care and take remedial action
Level VIII	> 10^–3^	Extremely high risk: mandatory to solve it
Cabbage (*Brassica oleracea*)	4.21 × 10^–3^	
Spinach (*Beta vulgaris*)	5.46 × 10^–3^	

**Figure 3:**
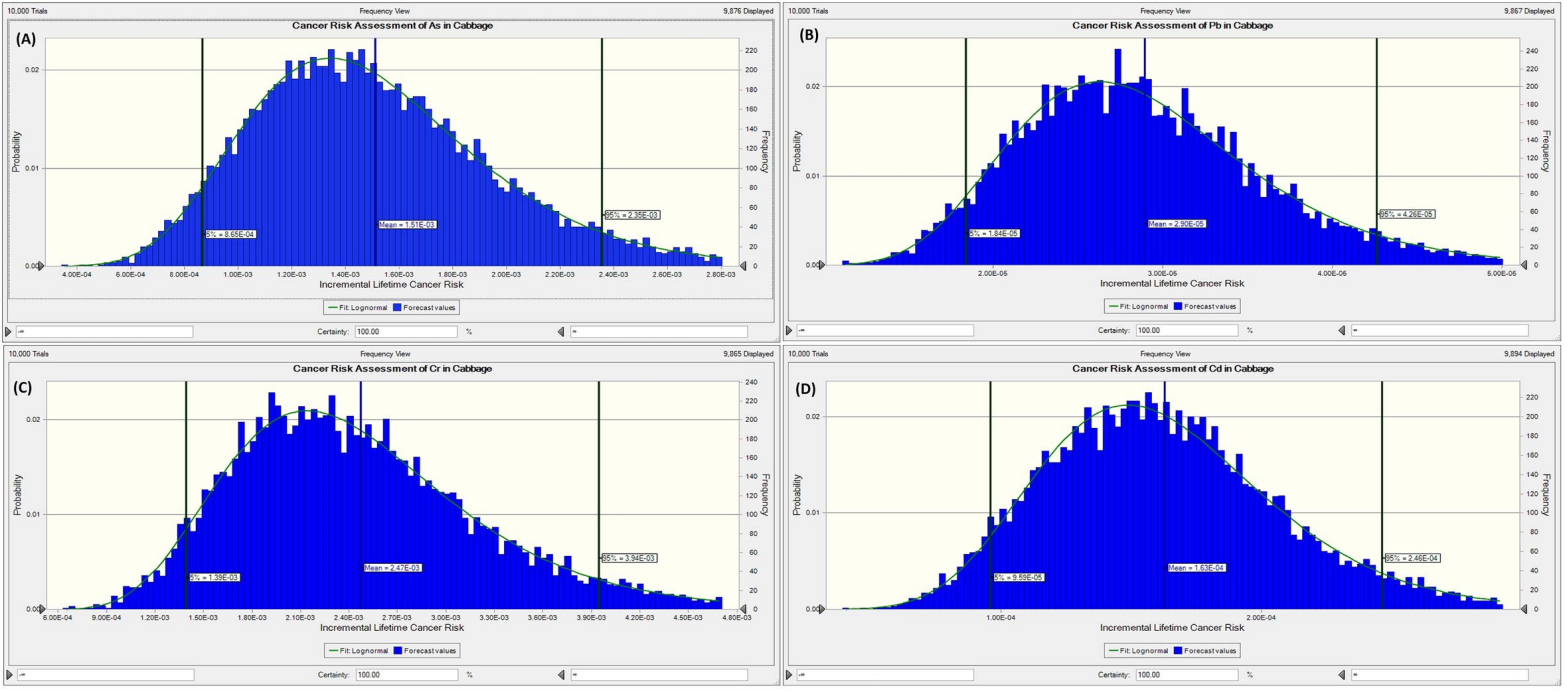
Probabilistic risk assessment for the potentially toxic metals in cabbage samples: (**A**) As, (**B**) Pb, (**C**) Cr, and (**D**) Cd.

**Figure 4:**
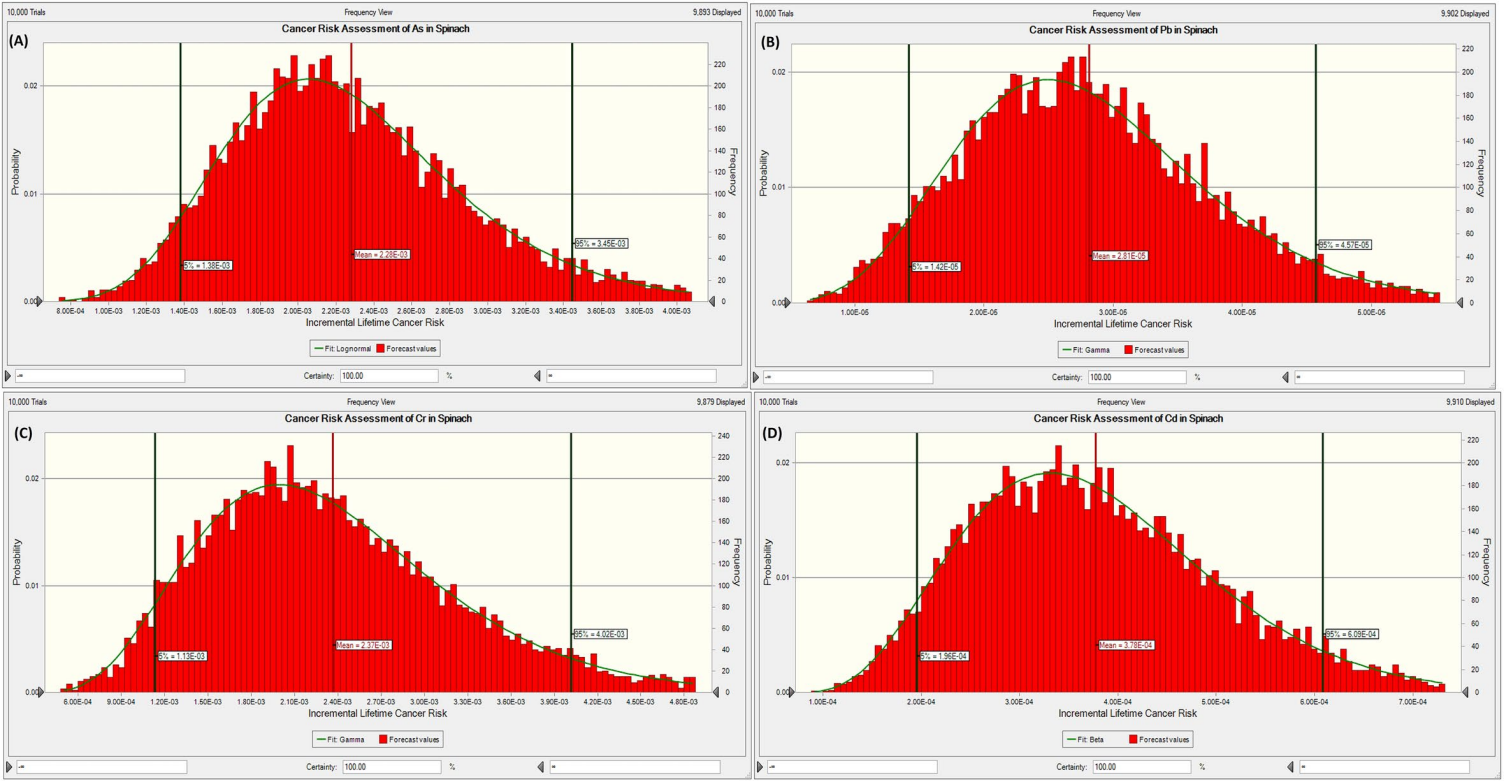
Probabilistic risk assessment for the potentially toxic metals in spinach samples: (**A**) As, (**B**) Pb (**C**) Cr, and (**D**) Cd.

**Figure 5:**
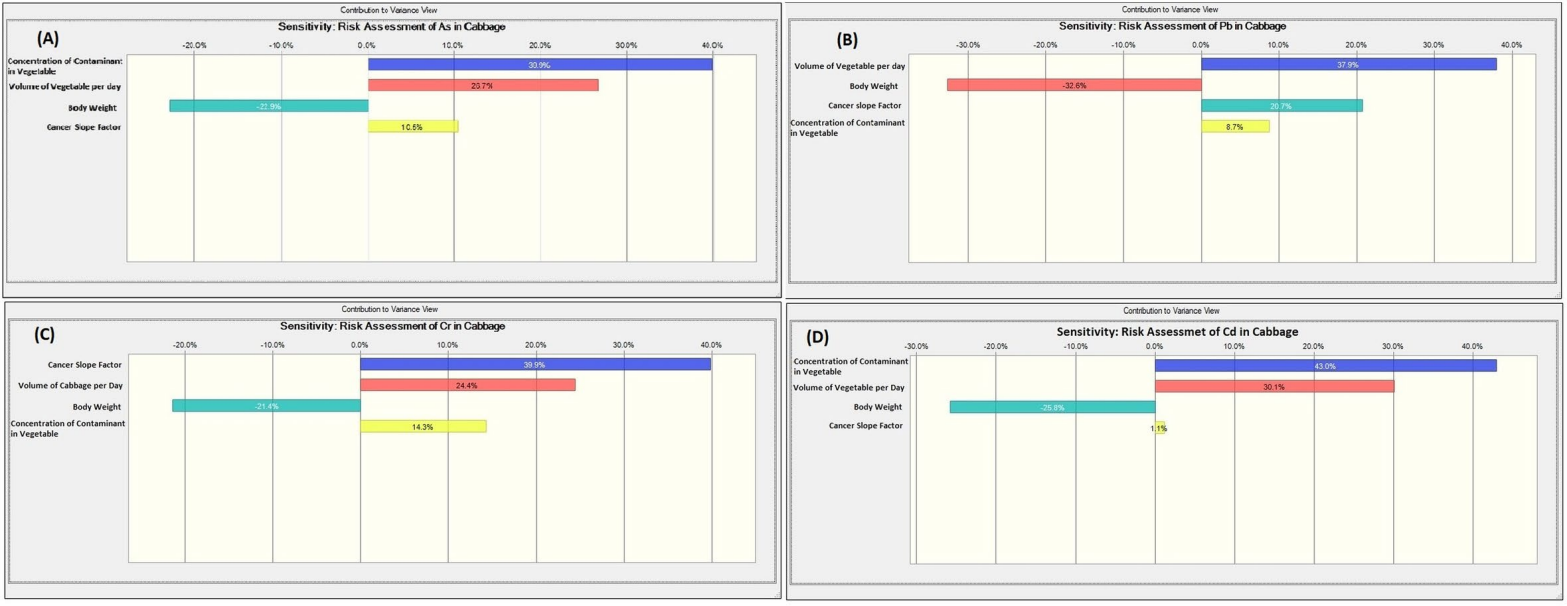
Sensitivity analysis for the potentially toxic metals in cabbage samples: (**A**) As, (**B**) Pb, (**C**) Cr, and (D) Cd.

**Figure 6:**
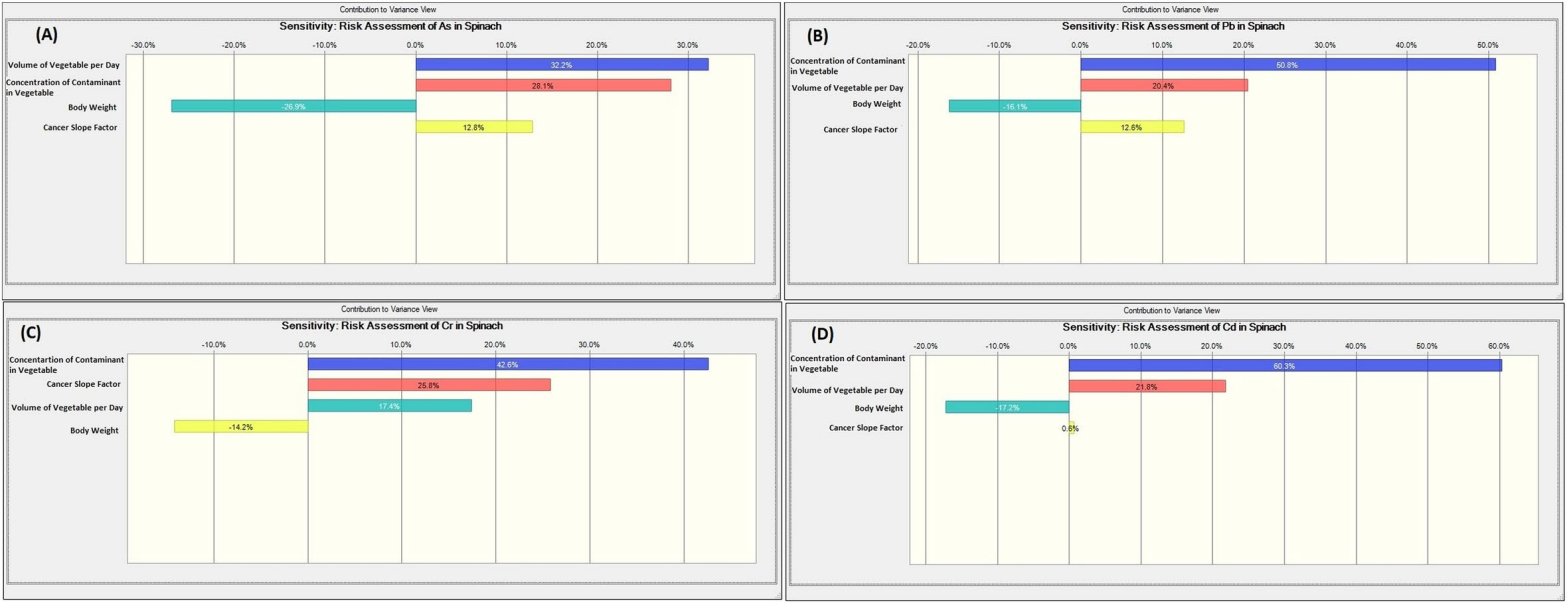
Sensitivity analysis for the potentially toxic metals in spinach samples: (**A**) As, (**B**) Pb, (**C**) Cr, and (**D**) Cd.

The above carcinogenic risks yield a single-point risk estimation. Hence, the MCs that provides a stochastic approach was broadly deployed. The simulation computed ten thousand trials. The 95^th^, 50^th^ (mean) and 5^th^ percentiles of the carcinogenic risk distribution and their respective sensitivity assessments for the vegetables, were evaluated and provided in Table 11 and Figs. 3, 4, 5 and 6.

**Table 11:** Summary of the probabilistic risk assessment for the vegetables using Monte Carlo simulation.

Vegetable	As			Pb			Cr			Cd			Cumulative		
	5%	Mean	95%	5%	Mean	95%	5%	Mean	95%	5%	Mean	95%	5%	Mean	95%
Cabbage	8.65E−4	1.51E−3	2.35E−3	1.84E−5	2.90E−5	4.26E−5	1.39E−3	2.47E−3	3.94E−3	9.59E−5	1.63E−4	2.46E−4	2.37E−3	4.17E−3	6.58E−3
Spinach	1.38E−3	2.28E−3	3.45E−3	1.42E−5	2.81E−5	4.57E−5	1.13E−3	2.37E−3	4.02E−3	1.96E−4	3.78E−4	6.09E−4	2.72E−3	5.06E−3	8.12E−3

The assessment of the least cancer risk (5^th^ percentile = best case scenario) for the cumulative risk for the cabbage shows that the general public has risk values higher than the acceptable range of 1.00E−4 and 1.00E−6. Likewise, the maximum probable cancer risk assessment (95th percentile = worst-case scenario) reveals values above the receptors' acceptable range. Similarly, the most likely risk estimation (50th percentile or mean) indicates that the most likely cancer risk for the cabbage is that four humans in the population of 1000 would be affected. For spinach, the assessment of the least cumulative cancer risk shows that the public has risk values higher than the acceptable range of 1.00E−4 and 1.00E−6 (i.e. 2.72E−3). The maximum probable cancer risk assessment for spinach exceeds the acceptable range for cancer risks. In the same way, the most likely risk estimation shows that the most likely cancer risks for spinach are that five humans in the population of 1000 would be affected. The result of the sensitivity analysis shows that the factor with the maximum impact on the carcinogenic risks caused by the consumption of the two vegetables from Ilorin, Nigeria was the concentration of the potentially toxic metals, followed by the volume of vegetables consumed, then the cancer slope factor while the body appears to have a negative correlation/contribution.

## Conclusion

The concentration of the potentially toxic metals determined was in the sequence Cr > Pb > As > Cd for soil samples thereby exceeding the WHO-recommended values in all cases. These higher values of toxic metals may be attributed to the redistribution of metal-bioavailability of ground tissue uptake in the aqueous phase. This pollution was corroborated by the modified pollution index and the enrichment factors (the pollution evaluation indices). The concentrations of the toxic metals in the irrigation water followed a similar pattern i.e. Cr > Pb > As > Cd. The irrigation water was neutral while the soil was slightly basic with both (soil and irrigation water) having conductivity values within the acceptable range. All the understudied toxic metals were found in the vegetables with Cr having the highest concentration followed by Pb and As, while Cd had the lowest concentration. The concentration of the toxic metals was higher in spinach than in cabbage. The higher toxic metal in spinach may be due to the soil geomorphology that enhances the geogenic impacts on roots-soil-metal retentive ability. This revelation was in agreement with the previous findings and was attributed to deposits of toxic metals on the leaves of spinach during irrigation. It was noted that the variation of the toxic metal contents in these vegetables is dependent on the physiochemical nature of the soil, and the vegetable's capacity to absorb each metal which is greatly affected by the environmental factors, planting, and farming methods, specie of plant, climatic conditions, etcetera. Expectedly, the Hazard Index (HI) value of spinach is higher than that of cabbage. This implies that spinach posesa higher non-carcinogenic risk. Most important is the fact that both the spinach and cabbage have HI values higher than 1 which is the standard set by USEPA. This follows that the general populace is at greater risk of non-carcinogenic risks for consuming this vegetable. Similarly, the value for the estimated cancer risks (ILCR) for spinach is higher than for cabbage. This also reveals that spinach poses a higher carcinogenic risk than cabbage. Worrisomely, the cancer risk for both vegetables exceeds the threshold range provided by USEPA. Similarly, the results of the Monte Carlo simulation revealed that the 5th percentile, 95th percentile, and 50th percentile of the cumulative probability of cancer risks due to the consumption of these vegetables exceeds the acceptable range of 1.00E−6 and 1.00E−4 recommended by USEPA. This, therefore, means that the probable risk of a cancerous effects is high and the public should be cautious as the risk level for both vegetables falls in Level VII (High Risk). Thus, the attention of the appropriate authorities is needed, and necessary remedial action is recommended using phytoremediation, phytostabilization, and phytoextraction for further research on the redistribution of toxic metals in a bioavailability aqueous controlled environment.

## Data Availability

The datasets used and/or analysed during the current study available from the corresponding author on reasonable request.
